# HIF1α activation in dendritic cells under sterile conditions promotes an anti-inflammatory phenotype through accumulation of intracellular lipids

**DOI:** 10.1038/s41598-020-77793-6

**Published:** 2020-11-30

**Authors:** Elizabeth G. Wood, Claire E. Macdougall, Hazel Blythe, Marc Clément, Romain A. Colas, Jesmond Dalli, Federica Marelli-Berg, M. Paula Longhi

**Affiliations:** 1 William Harvey Research Institute, Barts and the London School of Medicine and Dentistry, Queen Mary University of London, London, EC1M 6BQ UK; 2 INSERM U1148, Laboratory for Vascular Translational Science, Hôpital Bichat, 46 rue Henri Huchard, 75018 Paris Cedex, France

**Keywords:** Immunology, Adaptive immunity, Inflammation

## Abstract

Obesity is among the leading causes of elevated cardiovascular disease mortality and morbidity. Adipose tissue dysfunction, insulin resistance and inflammation are recognized as important risk factors for the development of cardiovascular disorders in obesity. Hypoxia appears to be a key factor in adipose tissue dysfunction affecting not only adipocytes but also immune cell function. Here we examined the effect of hypoxia-induced transcription factor HIF1α activation on classical dendritic cell (cDCs) function during obesity. We found that deletion of *Hif1*α on cDCs results in enhanced adipose-tissue inflammation and atherosclerotic plaque formation in a mouse model of obesity. This effect is mediated by HIF1α-mediated increased lipid synthesis, accumulation of lipid droplets and alter synthesis of lipid mediators. Our findings demonstrate that HIF1α activation in cDCs is necessary to control vessel wall inflammation.

## Introduction

Classical dendritic cells (cDCs) are the main antigen presenting cells and key regulators of immune homeostasis and immunity^1^. Constantly probing their surroundings, cDCs are poised to detect invading pathogens, initiate and direct adaptive immune responses. cDCs are found in peripheral tissue in an immature state, where they sense and sample the environment for self- and non-self-antigens. Under pathogen driven danger signals, e.g. toll-like receptor (TLR), cDCs undergo an intricate maturation process characterized by increased expression of co-stimulatory molecules, cytokine/chemokine production and enhanced migration to draining lymph nodes (dLN) to present antigen to naïve T cells^1^. In tissue, cDCs must adapt to specific metabolic cues such as nutrient restriction or low O_2_ tensions, which may modulate their biological functions. Indeed, in recent years it became apparent that disruption of key metabolic pathways can alter immune cell activation and differentiation.

Reduced oxygen or hypoxia, is a hallmark of multiple acute and chronic diseases and arises especially in inflamed tissues. Cellular adaptation to hypoxia rely on stabilization of the hypoxia-inducible factor-1 (HIF1) transcription factor, which drives the metabolic changes required for adaptation to hostile microenvironments. HIF1 is a heterodimer transcription factor consisting of a constitutively expressed β-subunit (HIF1β) and an oxygen-regulated α subunit (HIF1α)^2^. Under normoxic conditions, HIF1α is regulated through hydroxylation of proline residues, ubiquitination and rapid degradation by the proteasome. Under low oxygen concentrations, however, prolyl hydroxylase is inactivated and HIF1α can translocate into the nucleus for transcriptional regulation. HIF1α accumulation does not always occur under low O_2_ tension but can be triggered by oxygen-independent signalling events or cellular stress, such as toll-like receptor signalling and reactive oxidase species (ROS)—a phenomenon known as ‘pseudohypoxia’^3^.

Activation of the hypoxia pathway is believed to fuel inflammation, largely due to HIF1α activation in macrophages. Lipopolysaccharide (LPS) activation induces HIF1α protein accumulation in macrophages under normoxic conditions^4^ leading to metabolic reprogramming, succinate release, and IL-1β production^5^. HIF1α-deficient macrophages exhibit impaired inflammatory responses, with reduced glycolytic rate, motility and migration^6^. Similarly, toll-like receptor (TLR) activation in dendritic cells, leads to HIF1α stabilization, glucose uptake and glycolytic metabolic shift^7,8^. In this context, deletion of HIF1α results in decreased ATP production, cell survival and T cell priming^8–10^. However, the effect of HIF1α on dendritic cells function remains controversial. In other studies, particularly under hypoxic conditions, HIF1α activation has been shown to promote anti-inflammatory response^11,12^. HIF1α has pleiotropic effects regulating hundreds of genes encoding glycolytic enzymes, glucose transporters, matrix metalloproteinases, angiogenic and survival factors, that may depend on time and context. In addition, these divergent reports may in fact be explained by differences in experimental conditions or cell type targeted.

The present study was designed to investigate the role of HIF1α activation in cDCs in an experimental model of obesity by using mice with HIF1α deletion specifically in cDCs. Contrary to what was expected, deletion of HIF1α in cDCs promoted adipose tissue inflammation and atherosclerotic plaque formation. This effect was mediated by intracellular lipid accumulation and altered production of lipid mediators, which translated into reduced T cell stimulation.

## Results

### Deletion of *Hif1α* in cDCs leads to increased adipose tissue inflammation under high lipid and cholesterol diet

**Figure 1:**
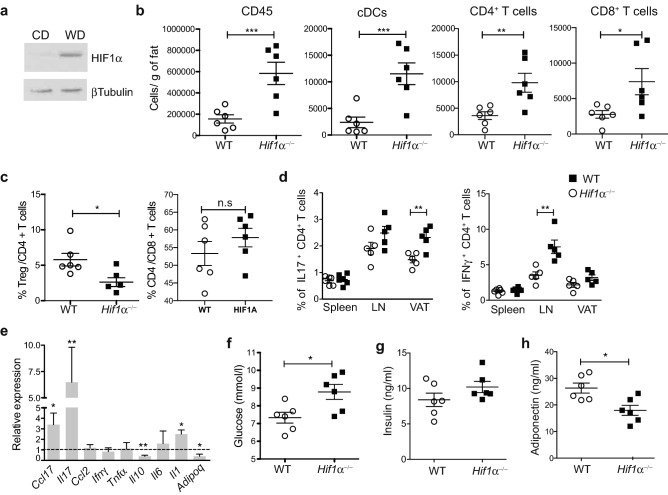
Activation of HIF1α in adipose-tissue cDC ameliorate obesity-induced adipose tissue inflammation. (**a**) Western blot image of HIF1α expression on purified VAT-cDCs. Uncropped image is shown in Suppl. Fig. S4. (**b**) VAT was digested from *Hif1α*^−/−^ and WT mice fed a WD for 12 weeks. Total numbers of CD45^+^ hematopoietic cells, cDCs, CD4^+^ and CD8^+^ T cells were quantified by flow cytometry (n = 6). (**c**) Graph indicate FoxP3^+^ Tregs/CD4^+^ and CD4^+^/CD8^+^ T cells ratios. (**d**) IL17 and IFNγ production by spleen, LN and VAT-infiltrated CD4^+^ T cell was evaluated by flow cytometry. Bars represent mean ± SEM (n = 5). (**e**) Quantitative RT-PCR analysis for gene expression in VAT from obese *Hif1α*^−/−^. Expression levels of all genes were normalized against GAPDH RNA and compared to WT mice, which was set as 1. Error bars indicate the geometric mean of 8 biological replicates. (**f**) Fasting glucose, (**g**) insulin and (**h**) adiponectin were detected by ELISA. Bars represent mean ± SEM (n = 6). Statistical analysis was performed with Student’s test or one-way ANOVA, *p < 0.05, **p < 0.01 and ***p < 0.0005.

Adipose tissue hypoxia has been causally implicated in obesity-induced inflammation and insulin resistance. Under lipid rich diet, adipocyte expansion decreases oxygen tension and ROS production, which leads to rapid activation of HIF1α^13,14^. cDCs are present in visceral adipose tissue (VAT) where they control tissue homeostasis^15^. To investigate the effect of HIF1α in cDCs under chronic, sterile inflammation, we generated cDC-specific *Hif1α* knockdown by crossing *Zbtb46*-CRE with *Hif1α*^fl/fl^ mice. As Zbtb46 is expressed in some non-haematopoietic cells, we performed mice chimeras where irradiated C57BL/6 mice were reconstituted with bone marrow cells from *Hif1α*^fl/fl ^× Zbtb46-CRE (referred to as *Hif1α*^−/−^) or *HIF1α*^fl/+ ^× Zbtb46-CRE (referred to as WT) mice. HIF1α deletion is shown in Suppl Fig. S1A. *Hif1α*^−/−^ or WT mice were fed a lipid-rich diet (western diet) for 12 weeks, which resulted in HIF1α increase in cDCs (Fig. 1a). Surprisingly, deletion of HIF1α in cDCs results in increased immune cell infiltration into visceral adipose tissue (VAT) from *Hif1α*^−/−^ mice, with increased numbers of cDCs, T cells and decreased regulatory T cells (Tregs) (Fig. 1b,c; Suppl Fig. S1b). Expression of the co-stimulatory molecule CD86 remained unchanged (Suppl Fig. S1c). Production of IL-17 in VAT-infiltrated T cells was increased in *Hif1α*^−/−^ compared to WT mice together with increased IFNγ production in lymph nodes (LN) (Fig. 1d; Suppl Fig. S1d). No differences were observed in splenic T cells (Fig. 1d). This contributes to the enhanced VAT inflammation in *Hif1α*^−/−^ mice as detected by RT-PCR (Fig. 1e).

Chronic inflammation in VAT plays a central role in the development of obesity-related insulin resistance and systemic metabolic abnormalities^16^. Thus, we evaluated if the increased inflammation in *Hif1α*^−/−^ mice could translate into systemic metabolic abnormalities. Body and VAT weight were similar between mice (Suppl Fig. S1e,f). *Hif1α*^−/−^ mice displayed a small increase in fasting glucose levels and serum insulin levels compared to WT mice, albeit this was not significant (Fig. 1g,h). Circulating concentrations of adiponectin were reduced in *Hif1α*^−/−^ mice indicative of adipocyte dysfunction (Fig. 1f–h). Despite signs of metabolic misbalance, *Hif1α*^−/−^ mice showed no sign of increased glucose and insulin tolerance during a glucose tolerance (GTT) and insulin tolerance (ITT) test (Suppl Fig. S1g,h). Thus, the results presented here indicate that inhibition of the Hif1 pathways under chronic, low oxygen conditions, promotes cDC activation.

### Deletion of *Hif1α* in cDCs accelerates the development of atherosclerosis

Obesity-induced chronic inflammation is tightly linked to the development of atherosclerotic plaque^17^. The atherosclerotic plaque is characterized by arterial wall thickening, ROS production and reduced oxygen supply in certain areas of the vascular intima creating a hypoxic milieu^18^. HIF1α expression was observed in macrophages and smooth muscle cells bordering the necrotic core, and was associated with inflammation and angiogenesis^19^. In addition, arteries are surrounded by perivascular adipose tissue which, under metabolic abnormalities, appears to promote vascular dysfunction and atherosclerosis^20^. Thus, we hypothesized that increased VAT inflammation in *Hif1α*^−/−^ mice might have a negative impact on the development of atherosclerosis. To test this, lethally-irradiated low density lipoprotein receptor knockout (*Ldlr*^*−/−*^) mice were reconstituted with bone marrow from *Hif1α*^fl/fl ^× Zbtb46-CRE (referred as *Hif1α*^−/−^ → *Ldlr*^−/−^) or HIF1α^fl/+^ × Zbtb46-CRE mice (referred as WT → *Ldlr*^−/−^). *Ldlr*^−/−^ mice, under fat rich diet, are known to show elevated plasma cholesterol (25 mmol/L) and rapid atherosclerotic plaque formation^21^.

**Figure 2:**
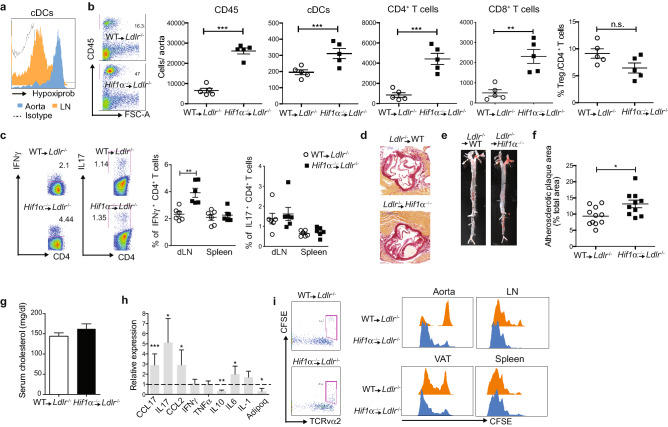
*Hif1α* deletion in cDCs promotes atherosclerotic plaque formation. *Hif1α*^−/−^ → *Ldlr*^−/−^ and WT → *Ldlr*^−/−^ mice were fed a WD for 12 weeks. (**a**) Flow cytometry analysis of aortic-infiltrated cDCs for Hypoxiprobe staining. (**b**) Aorta was enzymatically digested and the presence of CD45^+^ hematopoietic cells, cDCs, CD4^+^ T cells, CD8^+^ T cells and FoxP3^+^ Tregs were quantified by flow cytometry. Representative dot plot from aorta infiltrated CD45^+^ cells. Bars represent mean ± SEM (n = 5). (**c**) IFNγ and IL17 production was analysed by flow cytometry in spleen’s and dLN’s CD4^+^ T cell. Dot plot is representative of 3 independent experiments. Bars represent mean ± SEM (n = 6). (**d**) Representative Sirius red staining of aortic root. (**e**) Representative images of en face oil red O staining of atherosclerotic lesions in aorta and (**f**) quantification of atherosclerotic lesion size. Bars indicate mean ± SEM (n = 10). (**g**) Total cholesterol in obese mice (n = 10). (**h**) Quantitative RT-PCR analysis for gene expression in perivascular fat from obese *Hif1α*^−/−^. Expression levels of all genes were normalized against GAPDH RNA and compared to WT mice, which was set as 1. Error bars indicate the geometric mean of 7 biological replicates. (**i**) For in vivo antigen presentation, CFSE-labelled OTII cells were transferred i.v. one day before immunization with 200 μg OVA i.p. Three days later, frequency and division of proliferating OT-II T cells were analysed in aorta, LN, VAT and Spleen from Hif1α^−/−^ and WT mice by flow cytometry. Dot plots show gating strategy for CFSE^+^ OTII cells. Histograms show dilution of CFSE^+^ OTII gated cells and are representative of 3 independent experiments. Statistical analysis was performed with Student’s test, *p < 0.05, **p < 0.01 and ***p < 0.0005.

*Hif1α*^−/−^ → *Ldlr*^−/−^ and WT → *Ldlr*^−/−^ were fed a WD for 12 weeks, after which the presence of hypoxic cDCs in the atherosclerotic lesions was confirmed by pimonidazole staining (Fig. 2a). In agreement with increased VAT inflammation *Hif1α*^−/−^ → *Ldlr*^−/−^ mice had greater numbers of cDCs and T cells in atherosclerotic lesions compared to WT → *Ldlr*^−/−^ mice (Fig. 2b). The ratio of cDC subsets remains unchanged between *Hif1α*^−/−^ → *Ldlr*^−/−^ and control mice as well as the expression of CD86 (Suppl Fig. S2a,b). This was accompanied by enlarged draining lymph nodes (dLN) and increased T cell-mediated IFNγ production in *Hif1α*^−/−^ → *Ldlr*^−/−^ mice compared to controls (Fig. 2c; Suppl Fig. S2c). No differences were observed in the spleen (Fig. 2c; Suppl Fig. S2c). The numbers of LN migratory DCs (mDCs) and their CD86 expression remained constant (Suppl Fig. S2d,e). However, intracellular levels of IL12 were elevated in VAT-cDCs and mDC in *Hif1α*^−/−^ → *Ldlr*^−/−^ compared to control mice (Suppl Fig. S2f). Accordingly, *Hif1α* deficiency in cDCs was associated with a significant increase in atherosclerotic lesion development compared to control (Fig. 2d–f). Plasma cholesterol levels were comparable between the groups of mice (Fig. 2g). Similar to abdominal VAT, inflammatory markers in perivascular fat were increased in *Hif1α*^−/−^ → *Ldlr*^−/−^ compared to WT → *Ldlr*^−/−^ mice (Fig. 2h).

To confirm that this phenotype was a consequence of increased cDC activation, we tested cDC T cell stimulatory capacity in vivo. Enhanced proliferation of ovalbumin (OVA)-specific CD4^+^ T cells (OT-II) could be observed in the aorta, VAT and dLN but not spleen from *Hif1α*^−/−^ → *Ldlr*^−/−^ compared to control mice (Fig. 2i). Similarly, HIF1^−/−^ cDCs isolated from VAT an dLN of obese mice showed enhanced OT-II cell proliferation ex vivo compared to WT cDCs (Suppl Fig. S2g). OVA uptake was not affected by *Hif1α* deletion (Suppl Fig. S2h). Thus, depletion of HIF1α in cDCs under sterile conditions appears to unleash their activation.

### HIF1α induces intracellular lipid accumulation

Stabilization of HIF1α is known to promote aerobic glycolysis shifting the cells away from glucose oxidative phosphorylation. Upon TLR activation, HIF1α-dependent glycolytic switch enables macrophages and cDCs to produce metabolic intermediates and to fulfil cellular energetic demands required for full activation^22^ (Suppl Fig. S3a).

However, HIF1α has recently been shown to regulate mitochondrial glutamine metabolism (Suppl Fig. S3a). Glutamine once converted to α-ketoglutarate (αKG), can be either oxidized to succinate or reduced to isocitrate and then citrate by a mechanism known as reversed TCA cycle. The latter is favoured in cells where HIF1α is stabilized^23,24^.

**Figure 3:**
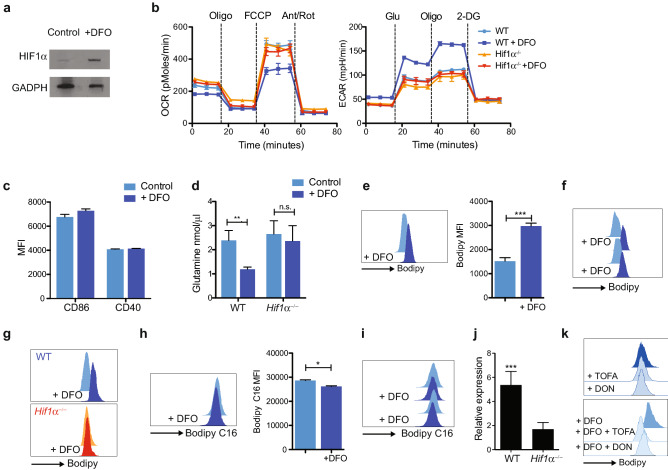
Hif1α activation induces intracellular lipid accumulation. (**a**–**k**) Bone-marrow cells were harvested from WT and Hif1α^−/*−*^ mice and cultured 7–8 days with recombinant Flt3L to obtain cDCs. Cells were then incubated 1 or 2 days with DFO to activate Hif1α. Uncropped image is shown in Suppl. Fig. S4. (**a**) Expression of Hif1α in cDCs detected by Western blot. (**b**) cDC were purified, counted and plated in a XF96 culture plate. O2 consumption rate (OCR) and medium acidification (ECAR) were measured in real time under basal conditions and in response to indicated inhibitors. Graph are representative of 4 independent experiments. (**c**) Graph indicate expression of CD86 and CD40 in untreated and DFO-treated cells (n-3). (**d**) Glutamine in medium was detected by ELISA (n = 5). (**e**) Intracellular neutral lipids were detected using Bodipy staining by flow cytometry (n = 3). Histogram is representative of 5 independent experiments. (**f**) Bodipy staining from in vivo DFO-treated cDCs. (**g**) Similar to F, but Bodipy stain was performed in control and DFO-treated *Hif1α*^−/*−*^ and WT cells. Histograms are representative of x independent experiments. (**h**) cDCs were incubated with Bodipy FL C16 to evaluate lipid uptake by flow cytometry (n = 3). (**i**) Bodipy FL C16 staining from in vivo DFO-treated cDCs. (**j**) Quantitative RT-PCR analysis for *Hilpda* expression from WT and *Hif1α*^−/−^ in cDCs upon DFO treatment. Expression level was normalized against GAPDH RNA and compared to untreated conditions, which was set as 1. Error bars indicate the geometric mean of triplicates. (**k**) As in e, but cells were pre-incubated with DFO and then treated with TOFA and DON overnight. Lipid accumulation was detected with Bodipy staining. Histograms are representative of 3 independent experiments. Statistical analysis was performed with Student’s test, *p < 0.05, **p < 0.01 and ***p < 0.0005.

In an attempt to understand the metabolic changes induced by HIF1α in cDCs, we moved to an in vitro system where cDCs were generated from bone-marrow with Flt3 ligand (Flt3L) and Hif1α was activated with desferrioxamine (DFO) (Fig. 3a; Suppl Fig. S3b),which facilitates HIF1 activation during seahorse assays. As expected, DFO induced a glycolytic switch characterized by reduced mitochondria O_2_ consumption (OCR) and increased extracellular acidification (ECAR) detected with the XF Mito Stress and the Glycolysis stress test respectively (Fig. 3b). This metabolic shift was HIF1α dependent (Fig. 3b) and equally affecting both main cDC subsets, cDC1 and cDC2 (Suppl Fig. S3c,d). DFO treatment alone did not affect expression of co-stimulatory molecules (Fig. 3c).

Despite a shift towards aerobic glycolysis, a functional electron transport chain and glutamine-derived carbon may still be required for cellular function. Indeed, in tumour cells, metabolic adaptation under hypoxia involves a switch from glucose to glutamine as a source of carbon for the synthesis of fatty acids^25^. We therefore tested glutamine consumption, which was indeed increased in DFO-treated compared to control cells (Fig. 3d).

During the reverse TCA cycle, glutamine-derived citrate can be transported to the cytoplasm to generate acetyl-CoA, necessary for anabolic reactions such as fatty acid synthesis. In tumour cells, HIF1α-induced uptake and reduction of glutamine induces lipid synthesis and storage in lipid droplets (LD). Interestingly, intracellular accumulation of lipids has been associated with poor cDCs activation and function in tumours^26^. Consistent with an increased glutamine uptake, DFO-treated cells exhibit increased intracellular lipid accumulation in vitro and in vivo as detected by bodipy staining (Fig. 3e,f). Similar results were observed with DMOG and hypoxia treatment (Suppl. Fig. S3e). DFO-induced intracellular lipid accumulation was HIF1α dependent (Fig. 3g). Uptake of C16-Bodipy (palmitate) was unchanged after DFO stimulation in vitro and in vivo suggesting that lipid droplets accumulation is not mediated by lipid uptake but rather lipid synthesis (Fig. 3h,i). Transcriptional levels of the hypoxia-inducible lipid droplet-associated protein (*Hilpda*), which is involved in intracellular neutral lipid deposition, were also elevated after DFO treatment (Fig. 3j).

To test if glutamine metabolism was responsible for lipid accumulation in cDCs, cells were treated with the fatty-acid synthase inhibitor TOFA and the glutamine antagonist DON. Inhibition of fatty-acid synthesis and glutamine usage, significantly reduced lipid droplets accumulation in DFO-treated cells (Fig. 3k). Collectively, these data suggest that HIF1α activation in cDCs stimulates fatty-acid synthesis and accumulation in lipid droplets.

### HIF1α-mediated lipid droplets accumulation suppresses cDC activation

**Figure 4:**
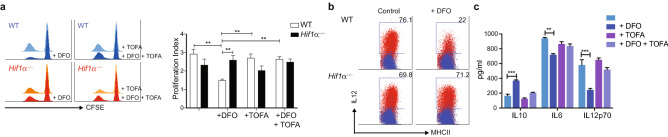
Hif1α activation impairs cDC function. (**a**) Antigen presentation capacity and activation of DFO-treated cDCs was evaluated by MLR. When indicated, cells were also treated with the fatty-acid synthesis inhibitor TOFA. T cell proliferation was assessed by CFSE dilution by flow cytometry. Histograms are representative of 3 independent experiments. Bar graph indicates proliferation index analysed by flow cytometry. (**b**) Cells were treated overnight with DFO and then stimulated with LPS for 6 h or overnight. Intracellular IL-12 production was evaluated by flow cytometry 6 h after stimulation. Dot plots are representative of 3 independent experiments. (**c**) Cytokines were measured in the supernatant WT cDCs after overnight stimulation by ELISA (n = 3). Statistical analysis was performed with one-way ANOVA, **p < 0.01 and ***p < 0.0005.

Accumulation of lipids in DCs has been shown to impair their antigen-presenting function in tumours. Mice with deleted expression of HIF1α on cDCs exhibit increased aortic and VAT inflammation. Thus, we tested whether DFO treatment could alter cDCs function in vitro*.* Indeed, DFO treatment significantly reduces cDCs stimulatory capacity in a mixed-leukocyte reaction (MLR). This effect was reverted after fatty-acid synthesis inhibition with TOFA (Fig. 4a). Similarly, pre-treating cells with DFO reduces cDC cytokine production after LPS treatment (Fig. 4b,c). Thus, similar to tumour-infiltrating DCs, lipid-laden cDC exhibit reduced immunogenicity.

### Lipid accumulation in cDCs alters the expression of lipid mediators

**Figure 5:**
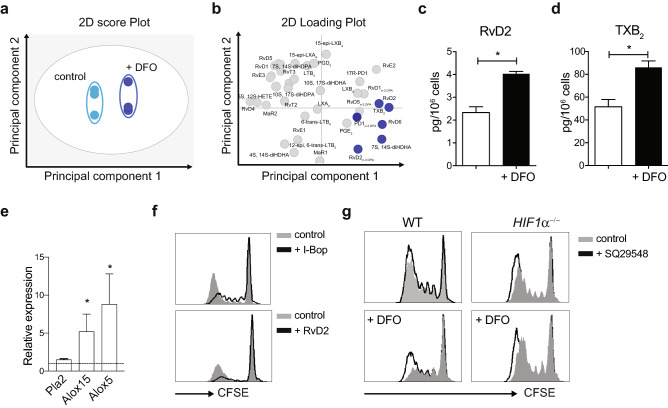
HIF1 stabilization induces changes in the cDC lipid mediator profile. DFO-treated cDCs cells were harvested for lipidomic analysis. Lipid mediators were extracted using solid-phase extraction techniques and identified using liquid chromatography-tandem mass spectrometry-based lipid mediator profiling. (**a**) 2-dimensional score plot of plasma LM-SPM and (**b**) corresponding 2-dimensional loading plot. Gray ellipse in the score plot denotes 95% confidence regions. (**c**) Bars representing significant increase of RvD2, and (**d**) TXB_2_ after DFO treatment (n = 3). (**e**) Quantitative RT-PCR analysis for early *Pla2* and late, *Alox15* and *Alox5*, enzymes involved in the synthesis of resolvins in VAT-cDCs from WT and *Hif1α*^−/−^ obese mice. Expression level was normalized against GAPDH RNA and compared to *Hif1α*^−/−^ cDCs, which was set as 1. Error bars indicate the geometric mean of triplicates. WT cDCs were pre-treated with DFO and then the TXA2 agonist I-Bop or RvD2 when indicated. cDC stimulatory capacity was evaluated by MLR. T cell proliferation was assessed by CFSE dilution by flow cytometry. Histograms are representative of 2 independent experiments. (**f**) As in E but T cell were cultured with the TP antagonist SQ29548. Statistical analysis was performed with Student’s test, *p < 0.05.

Neutral lipid stored in LD are mobilized by lipases to provide metabolic energy (through fatty acid oxidation) and lipids for membrane synthesis. However, recently, it has become evident that LD function as a major intracellular pool of arachidonic acid, which is the precursor for eicosanoid biosynthesis^27^. In addition, LD can often interact with peroxisomes apparently facilitating peroxisomal fatty acid oxidation^28^. To investigate a possible consequence of lipid accumulation and the synthesis of lipid mediators, we performed a lipid mediator profiling of DFO-treated and control cells (Fig. 5a,b). In these incubations, we identified mediators from all four major fatty acid metabolomes (Table S1). Production of prostaglandins and leukotrienes remained unchanged after DFO treatment, probably due to the absence of TLR stimulation (Table S1). However, incubation of these cells with DFO significantly increased resolvin D2 (RvD2) and thromboxane B_2_ (TXB_2_) a stable metabolite of TXA_2_ (Fig. 5c,d) concentrations. Accordingly, key enzymes contributing to the generation of resolvins were elevated in WT compared to HIF1^−/−^ VAT-cDCs from obese mice (Fig. 5e). RvD2 is a pro-resolving lipid-mediator that was recently shown to suppress T cell responses and to decrease TLR4 expression in monocytes^29^. Similarly, TXA_2_ modulates DC-T cell interaction and adaptive immune responses^30^. Interestingly, treatment with RvD2 or deletion of the TXA_2_ receptor, TP, significantly delays atherosclerosis progression^31^. Consistent with previous observations, treatment of cDCs-T cell cultures with RvD2 and TP agonist (I-Bop) reduces T cell proliferation in vitro (Fig. 5f). Similarly, the inhibitory effect of HIF1α activation in cDCs, was partially reverted with the TP antagonist SQ29548 (Fig. 5g). Thus, this data suggest that intracellular accumulation of lipids also impacts the production of bioactive lipid mediators by cDC.

## Discussion

The transcriptional factor HIF1α plays a central role in cellular adaptation to hypoxia. Physiologic O_2_ tension found in tissues varies between 2 and 9%. Hypoxic niches can be observed in a range of tissues including bone marrow, medulla of the kidney, intestinal epithelial and thymus^32–34^. Tissue oxygenation can be severely disrupted during pathological conditions such as obesity, solid tumours, stroke and inflammation leading to hypoxia and HIF1 pathway activation^35–37^. In obesity, HIF1 stabilization is propagated by mitochondrial ROS production^14^. In either of these conditions, HIF1α initiates a transcriptome response to help the cells adapt to a hostile environment. For example, HIF1α induces production of angiogenic factors to promote blood vessel formation; switches cellular metabolism to anaerobic glycolysis; reduces reactive oxygen species (ROS) and upregulates BCL-XL expression thus promoting cell survival^38,39^.

The link between HIF1 and inflammation has been increasingly appreciated over recent years. Inflammatory conditions are frequently characterized by tissue hypoxia, ROS production and HIF1α stabilization due to altered metabolic supply/demand ratios^40^. HIF1α activation in adipose tissue hypoxia is a triggering factor for obesity-induced inflammation^41^. Similarly, arterial wall thickening and inflammatory deposition in the atherosclerotic plaque leads to HIF1α stabilization, which appears to promote disease progression^18^. This pro-inflammatory effect appears to be partially mediated by HIF1α activation in myeloid cells. Indeed, mice with myeloid-specific deletion of HIF1α (via lysozyme M-Cre) exhibit improved glucose metabolism, reduced adipose tissue inflammation and decreased atherosclerotic plaque formation^42,43^. With this in mind, we investigated the implications of HIF1α activation in cDCs and its physio-pathological consequences in obesity-induced chronic inflammation.

In this study, we found that inhibition of HIF1α in cDCs promotes adipose tissue inflammation and accelerates the development of atherosclerosis. Deletion of HIF1α appears to unleash cDC activation with an increased in IL12 production and induction of IL17^+^ T cells. Not apparent difference on lymph node migration and phagocytosis could be detected but this is not an exhaustive study and other mechanisms may be involved. These findings oppose the anti-inflammatory effect observed in myeloid cells. Of note, HIF1 transcription has also been shown to activate multiple anti-inflammatory pathways such as the expression of arginase 1 and VEGF in M2-like tumor-infiltrating macrophages and the upregulation of A2B adenosine receptor promoting Th2 and Treg differentiation^44^. Therefore, it appears that HIF1 regulates cell effector function in a cell-dependent manner. Contrary to pro-inflammatory macrophages, cDCs in steady-state are poised to suppress immune responses in tissue, which may account for the phenotype observed. Indeed, cDC are important for the control of adipose tissue homeostasis while Flt3L-mediated expansion of cDCs protects mice from atherosclerosis^15,45^. Along those lines, expression of HIF1α in intestinal dendritic cells, which are crucial for the maintenance of mucosal immune homeostasis, suppresses colitis-induced inflammation^12^. These results suggest that activation of HIF1α facilitates cDCs cellular adaptation and survival, allowing them to pursue their anti-inflammatory function.

A key feature of the HIF1 transcriptional response is the upregulation of genes encoding glycolytic enzymes, glucose transporters and the pyruvate dehydrogenase kinase *Pdk1* thereby shunting pyruvate away from mitochondria^6^. Under these conditions, mitochondria are not supplied with glucose-derived acetyl-CoA, a central biosynthetic precursor for lipid synthesis. To compensate for this effect, recent studies in tumor cells have shown a complementary switch towards glutamine metabolism to support anabolism and growth under hypoxic conditions^46^. In cDCs, glutamine-derived lipid synthesis results in the accumulation of lipids in LDs. Interestingly, cDCs in solid tumors, which are in general hypoxic, are also characterized by LD accumulation and an anti-inflammatory phenotype^26^. Indeed, similar findings were described as a result of the ER stress response factor XBP1 activation in ovarian tumor-infiltrated cDCs^47^. XBP1 regulates HIF1α pathway activation and is essential for cell survival under hypoxic conditions^48^.

The mechanism by which lipid-laden DCs acquire a tolerogenic function is unclear. Recently it has been suggested that LD in tumor-DCs contain oxidatively truncated electrophilic lipids that interfere with cross-presentation^49^. However, the role of lipid mediators is not known. Lipid mediators are considered key signalling molecules in inflammation as they are locally produced through specific biosynthetic pathways in response to extracellular stimuli^50^. Once produced, they bind to their cognate G protein receptor to exert their function after which they are rapidly inactivated through specific processes^50^. Lipid mediators are involved in many physiological processes and their dysregulation has often been linked to various diseases including atherosclerosis^51^. Lipid mediators can be subcategorized into different groups, such as AA-derived eicosanoids (e.g. prostaglandins); and the newly identified anti-inflammatory and pro-resolving mediators derived from ω-3 polyunsaturated fatty acids such as resolvins^52^. They can have pro- or anti-inflammatory function: for example, pro-inflammatory lipid mediators, in general terms, include prostaglandins and leukotrienes, while pro-resolving mediators include lipoxins, resolvins, maresins and protectins^52^. The effect of several lipid mediators on DC function is well documented^34,53^, however, production of these mediators by cDC and its regulation remains uncharacterized. We here show that activation of HIF1 pathways promotes the expression of RvD2 and TXB_2_, two mediators involved in the regulation of T cell responses. The profile of lipid mediators is not only regulated at the enzymatic levels but also through substrate availability that may differ between tissues and physiological and pathological conditions^54^. Our data indicated that HIF1α activation can alter the production of lipid mediators through LD accumulation. How the lipid mediator profile is altered in tissue-cDCs in vivo will require further investigations.

There is currently an increasing interest in targeting immunometabolic pathways in inflammatory diseases and cancer. However, questions arise about the specificity and off-target effect of many metabolic drugs. Although deletion of HIF1α in cDCs results in increased obesity-tissue inflammation, therapeutic treatment with the selective HIF1α inhibitor PX-478 slows down the progression of obesity-induced adipose tissue inflammation^55^. This opposite effect can be mediated through inhibition of HIF1α activation in recruited inflammatory macrophages, inhibition of HIF1-dependent VEGF production or directly on adipocytes^56–59^. Thus, the use of HIF1α inhibitors should be evaluated with caution as it may increase the risk of obesity-induced inflammatory complications such as psoriasis and rheumatoid arthritis or risk of infections.

## Methods

### Mice

B6.129s7-*Ldlr*^*tm1Her*^/J (Ldlr-KO), B6.129-*Hif1a*^*tm3Rsjo*^/J (HIFa^loxp^) and B6.Cg-Tg(TcraTcrb)425Cbn/J (OT-II) mice were purchased from Jackson Laboratory (US); C57BL/6 were purchased from Charles River laboratories (UK); Zbtb46-Cre^+^ mice were kindly provided by Nussenzweig (The Rockefeller University, NY). As previously described^15^, mice were housed in temperature- and humidity-controlled rooms at 22 °C and 55% humidity, with a 12 h light/12 h dark cycle. Mice were fed Chow or Test Diet AIN-76A (Test Diet IPS Ltd) and given water ad libitum; animals were rehoused in clean cages weekly. 15 g of Z-NEST (IPS Ltd) was used as nesting material to help regulate temperature and light levels. For chimeras, Ldlr-KO male mice were γ-irradiated twice with 500 rad 3 h apart. Three hours later, mice were reconstituted with marrow cells (3 × 10^6^) that had been harvested from the femurs and tibias of *Hif1α*^−/−^ and control WT littermates. Mice were maintained on acidified water during the critical 4 weeks reconstitution period. Mice fed Western diet that failed to gain more than 20% of body weight were excluded from the study. All animal experimental protocols were carried out in accordance with UK government Home Office licensing procedures and regulations, and approved by QMUL’s Animal Welfare and Ethical Review Board (AWERB-7007443).

### Flow cytometry staining

VAT was collected from euthanised mice, from the mesenteric, retroperitoneal and abdominipelvic fat depot sites. The tissue was weighed and digested with 2370 U/ml Collagenase II (Sigma) and 23 U/ml DNase (Sigma)/gram of VAT. Immune cells present in the vascular fraction were obtained after centrifugation and lysed of red blood cells before staining. For aortic-single cell preparation, perivascular fat and cardiac muscle was carefully removed and aorta isolated under a dissecting microscope. Aortic segments, including aortic sinus, aortic arch and thoracic aorta were digested with 607.5 U/ml collagenase I, 187.5 U/ml collagenase XI, 90 U/ml hyaluronidase and 90 U/ml DNase in Hank’s balanced salt solution for 1 h at 37 °C. Single cell suspensions were stained with fixable Aqua dead cell stain (Invitrogen) to exclude dead cells from analysis and surface markers; DX5-FITC, CD16/32-FITC, F4/80-PerCP, CD206-BV421, CD11c-BV605, CD11c-PE-CY7, CD103-APC, MHCII-AF700, CD11b-AF780, MertK-PE, CD45-PE-CF594, CD64-PE-Cy7, CD8-FITC, Ly6G-BV421, NK1.1-BV605, CD3-AF700, CD3-BV421, CD86-PE, CD4-PE-Cy7, CD4-PeCP-Cy5.5, B220-PerCP, B220-FITC (eBioscience/BioLegend/BD Bioscience/R&D/ThermoFisher). Samples were stained at 4 °C for 30 min and fixed at 4 °C for 30 min with 1% PFA. For intracellular staining of FOXP3-PE (Biolegend), samples were incubated in permeabilization/fixation buffer (eBioscience) at 4 °C for 30 min and washed in permeabilization buffer before staining with fluorescently conjugated primary antibody at 4 °C for 30 min in permeabilization buffer. For hypoxia studies, mice were injected i.p. with 60 mg/kg bw of Hypoxiprobe™-1 (Pimonidazole Hydrochloride, Hypoxiprobe). Two hours later, aortas were harvested, digested and stained with anti-pimonidazole monoclonal antibody-PE (Hypoxiprobe). For intracellular cytokine analysis, immune cells were isolated from tissues and stimulated with 20 ng/ml Phorbol 12-myristate 13-acetate (PMA) (Sigma) and 1 µg/ml Ionomycin (Sigma) for 6 h, in the presence of 10 µg/ml Brefeldin A (Sigma) for the last 5 h. For intracellular staining, samples were first fixed with BD Cytofix/Cytoperm kit (BD Bioscience) and stained for 15 min with IFNγ-APC and IL-17-PE antibodies (Biolegend) following manufacturer’s instructions. Samples were then analysed by flow cytometry using a LSR Fortessa (BD Biosciences) and FlowJo version 10 software.

### DC innate and adaptive responses

cDC allostimulatory capacity was evaluated in vitro. For this, bone-marrow (BM) cells were harvested from *Hif1α*^−/−^ and WT mice injected previously with B16-Flt3l and cultured in the presence of 0.2 µl/ml recombinant Flt3 ligand (Invitrogen) for 8 days. Cells were then treated with 250 µM DFO (Desferrioxamine, Sigma) overnight. Alternatively, BM-cDCs were treated with DFO for 2 days and 1.2 µM TOFA (5-(Tetradecyloxy)-2-furoic Acid, Sigma) for the last 24 h. CD4^+^ T cells were harvested from the spleen of BALBc mice by CD4^+^ bead positive selection (MACS Miltenyi Biotec) and labelled with CFSE 3 μM (Invitrogen). Cells were mixed at a ratio of 1:5 (5 × 10^4^ CD11c^+^ cDCs: 2.5 × 10^5^ CD4^+^ T cells/well) and incubated for 3–5 days. When applicable, 10 nM of RvD2 (Cayman Chemicals) produced in house, 1 μM of I-BOP ([1S-(1α,2β(5*Z*),3α(1*E*,3S^*^),4α)]-7-[3-(3-hydroxy-4-(4′-iodophenoxy)-1-butenyl)-7-oxabicyclo-[2.2.1]heptan-2-yl]-5-heptenoic acid) or 1 μM of SQ-29548 ([1S-[1α,2α(Z),3α,4α]]-7-[3-[[2-[(phenylamino)carbonyl]hydrazino]methyl]-7-oxabicyclo[2,2,1]hept-2-yl]-5-heptenoic acid,) were added to the cultures. T cell division was assessed by CFSE dilution by flow cytometry and cytokine production in the supernatant by ELISA.

For in vivo cDC T cell stimulatory capacity, mice were injected intravenously with ovalbumin-specific OTII cells purified from spleen and lymph nodes by CD4^+^ bead positive selection (MACS Miltenyi Biotec) and labelled with CFSE 3 μM (Invitrogen). The next day, mice were immunized with 200 μg of Ovalbumin (Sigma) i.p. Three days later, immune cells were isolated from spleen, lymph nodes, aorta and visceral adipose tissue and OT-II cell division was evaluated by CFSE dilution by flow cytometry. Alternatively, mice were immunized with 200 μg of Ovalbumin (Sigma) i.p. and cDCs from VAT and dLN were isolated four hours later, purified by cell sorting and cultured with CFSE-labelled OT-II cells at a ratio of 1:5 in vitro. CFSE dilution was evaluated by flow cytometry. To evaluate ovalbumin uptake, mice were injected i.p. with 10 μg of OVA-Alexa Fluor-555. Two hours later, visceral adipose tissue was harvested and digested. Ova uptake by cDCs was analysed by flow cytometry.

BM-cDCs were treated with DFO overnight. Cells were then plated at 10^6^ cells/well and stimulated with 20 ng/ml LPS (sigma) in the presence of DFO. After culturing overnight, the supernatant was collected and levels of IL-10, IL-6 and IL-12p70 were determined by murine ELISA (eBioscience). Alternatively, cells were stimulated with LPS for 6 h and in combination with 10 µg/ml Brefeldin A (Sigma) for the last 5 h. Intracellular expression of IL-12 was assessed by flow cytometry as previously described. For in vivo IL-12 production, mice were immunized with 1 μg of LPS (Sigma), 2 h later, tissues were harvested, digested and incubated with 10 µg/ml Brefeldin A (Sigma) for an additional 4 h.

### Western blotting

Western blotting was performed as describe in^15^. Protein lysates were prepared from purified cDCs using RIPA buffer with phosphatase and protein inhibitors (Pierce). Proteins were separated with SDS-PAGE and transferred to Immobilon™ PVDF membrane (Millipore). Membranes were blocked for 1 h at room temperature in PBST containing 5% (w/v) milk, incubated overnight at 4 °C with anti-Hif1α antibody (Cell signalling) and subsequently with HRP-conjugated secondary antibody (Amersham Bioscience). Blotted proteins were detected using an EZ-ECL™ HRP chemiluminescence detection kit (Biological Industries) and exposed on to Hyperfilm™ photo film (Amersham).

### Quantitative Real-time PCR

RT-PCR was performed as previously described^15^. Briefly, RNA from 100 mg VAT was isolated using RNeasy Lipid Tissue Mini Kits (Qaigen) following the manufacturer’s instructions. Reverse transcription to cDNA was performed using High-Capacity RNA-to-cDNA Kits (Applied Biosystems) and stored at − 80 °C. Gene expression was performed using SYBR Green Supermix (Bio-Rad), according to the manufacturer’s instructions, and analysed using a CFX connect light cycler (Bio-Rad). Gene-relative expression was calculated using the ΔΔCT method and normalised to a reference control (GAPDH) with control sample set as 1. Average ratio from replicates was calculated using geometric mean. The following primers were used for the analysis (Invitrogen, UK);

- IL10_forward: AAA CAA AGG ACC AGC TGG AC
- IL10_reverse: TTC CGA TAA GGC TTG GCA AC
- TNFα_forward: TCG TAG CAA ACC ACC AAG TG
- TNFα_reverse: TTT GAG ATC CAT GCC GTT GG
- IL6_forward: TAG TCC TTC CTA CCC CAA TTT CC
- IL6_reverse: TTG GTC CTT AGC CAC TCC TTC
- IL1_forward: AAC CTG CTG GTG TGT GAC GTT C
- IL1_reverse: CAG CAC GAG GCT TTT TTG TTG T
- INFγ_forward: CAG CAA CAG CAA GGC GAA A
- INFγ_reverse: CTG GAC CTG TGG GTT GTT GAC
- IL17_forward: AAA GCT CAG CGT GTC CAA AC
- IL17_reverse: TTC TGG AGC TCA CTT TTG CG
- CCL2_forward: GCT GGA GCA TCC ACG TGT T
- CCL2_reverse: ATC TTG CTG GTG AAT GAG TAG CA
- CCL17_forward: GGA TGC CAT CGT GTT TCT GA
- CCL17_reverse: GCC TTC TTC ACA TGT TTG TCT TTG
- Alox15_forward: GAC ACT TGG TGG CTG AGG TCTT
- Alox15_reverse: TCT CTG AGA TCA GGT CGC TCCT
- Alox5_forward: TCT TCC TGG CAC GAC TTT GCT G
- Alox5_reverse: GCA GCC ATT CAG GAA CTG GTAG
- Pla2g4a_forward: GAT GAG GCT CAA GGA CCC AAA G
- Pla2g4a_reverse: GAA TAA AGC CGA GTC GCT CAC C
- Apidoq_forward: GAC GTT ACT ACA ACT GAA GAG C
- Apidoq_reverse: CAT TCT TTT CCT GAT ACT GGT C
- GAPDH_forward: GGC TCA TGA CCA CAG TCC A
- GAPDH_reverse: CAC ATT GGG GGT AGG AAC AC

### Whole mount aorta and aortic root staining

Immediately after euthanisation, the WD fed mice were flushed free of blood with cold PBS. Using a dissection microscope, the heart and aorta, including the arch with its branching vessels, thorax and abdominal section, were carefully excised and attached fat removed. The aorta was cut away from the heart just above the aortic synapse and both fixed in 4% PFA overnight. The aorta was opened longitudinally for en face Oil Red O staining. To prepare the stain 3 parts 5 mg/ml Oil Red O solution in isopropanol was diluted with 2 parts distilled water and allowed to solubilise for 1 h prior to 0.45 μm filtration. The aortas were washed in distilled water then transferred into freshly prepared Oil Red O solution for 15 min at room temperature (RT). They were then washed in 60% isopropanol for 5 min. This was repeated until no more red colour came out into the wash. Then the aortas were transferred into distilled water. Glycerol gelatin (Sigma) was used to mount the aortas onto slides and they were visualised using a M205 FA microscope (Leica). Images of the aortas were analysed blindly using ImageJ to quantify the percentage lesion density. The top half of the hearts were paraffin embedded, sectioned and mounted onto slides ready for Sirius Red staining by the BCI Pathology Core Service. The sectioned were visualised using a Pannoramic 250 High Throughput Scanning Microscope.

### Lipid mediator profiling

Lipid profiling was performed by QMUL lipidomic facility as described in^60^. Cell incubations were placed in 2 volumes of ice-cold methanol (Thermo Fisher Scientific) containing internal standards (d_8_-5*S*-hydroxy eicosatetraenoic acid, d_5_-RvD2, d_5_-LXA_4_, d_4_-PGE_2_, and d_4_-leukotriene LTB_4_ (all from Cayman Chemical); 500 pg each were added to the sample. LMs were extracted and identified as described previously^60^. Briefly, the samples in methanol were incubated for 45 min at − 20 °C for protein precipitation, and centrifuged at 1900*g* at 4 °C for 10 min. The methanol content of the supernatant was evaporated to less than 1 ml using nitrogen gas stream, and the LMs were extracted with automated Extra-Hera system (Biotage, Uppsala, Sweden) employing solid-phase extraction. The methyl formate eluates were concentrated and injected to liquid chromatography-tandem mass spectrometry (LC–MS/MS) system (LC-20AD HPLC (Shimadzu) and SIL-20AC autoinjector (Shimadzu) paired with QTrap 6500 + (ABSciex, Framingham, MA, USA). LMs were identified and quantified using multiple reaction monitoring the parent (Q1) and daughter (Q3) ions in negative ionization mode. Identification was conducted in accordance with published criteria, matching the retention time with authentic and synthetic standards (from Cayman Chemical, prepared in house, or provided by Charles N. Serhan, Harvard Medical School, Boston, MA, USA) and identifying at least 6 diagnostic ions from the MS/MS spectra PMID: 29363065.

### Metabolic assays

Adiponectin, Insulin (Merk Millipore), and total cholesterol (Abcam) were measured in the plasma from fasted mice by ELISA. Glucose metabolism was assessed by Glucose (GTT) and Insulin (ITT) tolerance tests after 6 h fasting as previously described in^15^. For GTT, mice were administered with 1.5 mg d-glucose/g of body weight (Sigma) or 0.5 U insulin/kg of body weight (Actrapid) via intraperitoneal injection for ITT. In both cases, blood glucose measurements were taken from tail bleeds at 15, 30, 60, 90, 120, 180 min after injection using blood glucose meter and test strips (FreeStyle Optium Neo, Abbott). Body weight and food intake was recorded weekly. Weight of total visceral adipose tissue harvested was recorded for cell number calculations and to determine the percentage of body weight.

To test mitochondrial respiration and glycolysis, BM-cDCs were treated with DFO overnight and plated at 2 × 10^5^/well onto Seahorse XF96 cell plates. Mito stress and Glycolysis stress test were performed with the Seahorse XF96 Extracellular Flux Analyzer (Agilent) flowing manufacturer’s instructions. Glutamine concentration was evaluated in supernatant from overnight DFO-treated BM-cDCs by ELISA (Abcam). For intracellular lipid detection, BM-cDCs were incubated with Bodipy FL or Bodipy-C16 (Invitrogen) at RT for 15 min in PBS. For ex vivo staining, mice were injected 50 μg/g bw DFO i.p. for two consecutive days Mice were euthanised the following day and spleens were digested with 400 U/ml Collagenase D (Roche). Splenocytes were stained with Bodipy FL or Bodipy-C16 as explained above.

### Statistical analysis

Statistical analysis was carried out with Graph Prism 8 (GraphPad Software LLC) and significance was evaluated by Student’ two tailed *t* test. For GTT and ITT, statistical significance was evaluated with 2-way ANOVA followed by post-hoc Bonferroni test. Differences within means were significant when *P* values of 0.05 or less. Normality was assessed using the Kolmogorov–Smirnov and Shapiro–Wilk test. Data are represented as mean and standard deviation.

## Supplementary information

Supplementary Information.
