# Supplementary material for: HIF1α activation in dendritic cells under sterile conditions promotes an anti-inflammatory phenotype through accumulation of intracellular lipids
Source: Sci Rep. 2020 Nov 30;10:20825. doi: 10.1038/s41598-020-77793-6 (PMC7705732; doi:10.1038/s41598-020-77793-6)
Supplement: Supplementary file 1 — Supplementary Information. [file 41598_2020_77793_MOESM1_ESM.docx]

**Supplemental Materia**

**HIF1α activation in dendritic cells under sterile conditions promotes an anti-inflammatory phenotype through accumulation of intracellular lipids.**

**Authors:** Elizabeth Wood^1^, Claire E. Macdougall^1^, Hazel Blythe^1^, Marc Clément^2^, Romain A. Colas^1^, Jesmond Dalli^1^, Federica M. Marelli-Berg^1^, M. Paula Longhi^1^†

**Affiliations:**

^1^William Harvey Research Institute, Barts and the London School of Medicine and Dentistry, Queen Mary University of London, London, EC1M 6BQ, UK.

^2^ INSERM U1148, Laboratory for vascular translational science, Hôpital Bichat, 46 rue Henri Huchard, 75018 Paris Cedex, France.

**Supplemental Figures**

**Figure S1. Unchanged glucose and insulin sensitivity in *Hif1a^-/-^* mice.** (a) Western blot image of HIF1α expression on purified VAT-cDCs from obese WT and *Hif1a^-/-^* mice. Uncropped image is shown in Suppl. Figure S4. (b) VAT gating strategy. (c) Bar graph represent CD86 expression of VAT-cDCs from obese mice. (d) Representative dot plots showing IL17 and IFNγ production by VAT-infiltrated CD4^+^ T cell. (e) Body weight *Hif1α^-/-^* and WT mice fed WD for 12 weeks. (f) VAT content was calculated as percentage of body weight. Graphs represent the mean ± SEM and are representative of 2 independent experiments. (g-h) Whole body glucose homeostasis measured in (*Hif1α^-/-^* and WT mice on WD by intraperitoneal glucose tolerance tests (GTT; n=8) and insulin tolerance tests (ITT; n=8). Statistical significance at different time points analysed by 2-Way-ANOVA with Bonferroni's post-test.

**Figure S2. *Hif1α* deletion promotes cDC activation.** *Hif1α*^-/-^→ *Ldlr*^-/-^ and WT→ *Ldlr*^-/-^ mice were fed a WD for 12 weeks. (a) cDC2/cDC1 ratio in the aorta. (b) CD86 expression in aortic cDC subsets. (c) IFNγ and IL17 production was analysed by flow cytometry in spleen’s and dLN’s CD4^+^ T cell (n=6). (d) Gating strategy for resident (rDCs) and migratory (mDCs) cDC in lymph nodes. Graphs represent percentage and total numbers of rDCs and mDCs in dLN. (e) CD86 expression in rDCs and mDCs. (f) IL12 production was analysed *ex-vivo* in cDCs by flow cytometry. Dot plots show representative gating strategy for cDCs defined as CD64^-^CD16^-^CD19^-^B220^-^DX5^-^CD3^-^CD11c^+^MHCII^+^. Graph represents the percentage of IL12+ cDC (n=4). (g) *Ex vivo* OT-II stimulatory capacity by VAT-cDCs and dLN mDCs. T cell proliferation was evaluated by CFSE dilution. (h) Ovalbumin-A555 uptake by VAT-cDCs in vivo after i.p. injection. Statistical analysis was performed with Student’s test or one-way ANOVA when applicable, *p<0~~.~~05, **p<0~~.~~01 and ***p<0.0005.

**Figure S3. Hif1α activation induces a metabolic switch in cDC subsets.** (a) Diagram depicting mitochondria TCA cycle. The reactions regulated by of HIF1α activation are in red; i) inhibition of pyruvate entry to mitochondria and ii) glutamine uptake and reduction towards citrate conversion. (b-c) WT and Hif1α^-/^*^-^* mice were injected with B16-Flt3L subcutaneously. After 10 days, bone-marrow cells were harvested from mice and cultured for 7-8 days with recombinant Flt3L to obtain cDCs. (b) Dot plots show BM-cDC cells phenotype. (c) Dot plots show gating strategy for BM-cDC sorting identified as Live CD64^-^B220^-^CD11c^+^MHCII^+^ cells. (d) cDC1 and cDC2 were purified by cell sorting as indicated in (c) and plated in a XF96 culture plate. O_2_ consumption rate (OCR) and medium acidification (ECAR) were measured in real time under basal conditions and in response to indicated inhibitors. Graph are representative of 2 independent experiments. (e) Bone-marrow cells were harvested from WT and Hif1α^-/^*^-^* mice were incubated 48 h with DMOG or at 1% O_2_ to activate Hif1α. Intracellular neutral lipids were detected using Bodipy staining by flow cytometry (n=3). Graph indicate Bodipy mean fluorescence of untreated, DMOG-treated and hypoxic cDCs.

| DHA bioactive metabolome | Q1 | Q3 | Control | DFO |
| --- | --- | --- | --- | --- |
| RvD1 | 375 | 215 | 0.59 ± 0.08 | 0.27 ± 0.23 |
| RvD2 | 375 | 215 | 2.33 ± 0.44 | 4.23 ± 0.18** |
| RvD3 | 375 | 147 | - | - |
| RvD4 | 375 | 101 | 5.97 ± 0.10 | 7.48 ± 0.39 |
| RvD5  RvD6 | 359  359 | 199  159 | 5.89 ± 0.36  0.84 ± 0.07 | 5.20 ± 0.48  1.31 ± 0.10 |
| 17R-RvD1  17R-RvD3 | 375  375 | 233  147 | 2.47 ± 0.56  - | 2.04 ± 0.09  - |
|  |  |  |  |  |
| PD1 | 359 | 153 | - | - |
| 17R-PD1 | 359 | 153 | 0.55 ± 0.12 | 0.68 ± 0.02 |
| 10S,17S-diHDHA | 359 | 153 | 6.00 ± 0.33 | 5.99 ± 0.67 |
| 22-OH-PD1 | 375 | 153 | - | - |
|  |  |  |  |  |
| MaR1 | 359 | 177 | 1.44± 0.37 | 2.14 ± 0.93 |
| MaR2 | 359 | 191 | 1.56 ± 0.21 | 1.81 ± 0.02 |
| 22-OH-MaR1 | 375 | 221 | - | - |
| 14-oxo-MaR1 | 357 | 248 | - | - |
| 7S, 14S-diHDHA | 359 | 221 | 3.87 ± 0.98 | 7.90 ± 1.02* |
| 4S, 14S, diHDHA | 359 | 101 | 2.98 ± 0.19 | 2.65 ± 0.31 |
|  |  |  |  |  |
| n-3 DPA bioactive metabolome |  |  |  |  |
| RvT1 | 377 | 211 | - | - |
| RvT2 | 377 | 197 | 1.06 ± 0.34 | 0.71 ± 0.16 |
| RvT3 | 377 | 173 | 4.66 ± 0.08 | 3.75 ± 0.85 |
| RvT4 | 377 | 211 | 4.61 ± 0.51 | 4.21 ± 0.38 |
|  |  |  |  |  |
| RvD1_n-3 DPA_ | 377 | 143 | 2.96 ± 0.32 | 4.14 ± 0.57 |
| RvD2_n-3 DPA_ | 377 | 261 | 0.49 ± 0.06 | 0.75 ± 0.27 |
| RvD5_n-3 DPA_ | 361 | 199 | 8.07 ± 0.45 | 14.41 ± 1.48* |
|  |  |  |  |  |
| PD1_n-3 DPA_ | 361 | 155 | 0.17 ± 0.11 | 0.30 ± 0.08 |
| 10S, 17S-diHDPA | 361 | 155 | 7.58 ± 0.71 | 6.77 ± 0.70 |
|  |  |  |  |  |
| MaR1_n-3 DPA_ | 361 | 223 | - | - |
| 7S, 14S-diHDPA | 361 | 223 | 22.78 ± 1.79 | 30.66 ± 2.18* |
|  |  |  |  |  |
| EPA bioactive metabolome |  |  |  |  |
| RvE1 | 349 | 161 | 9.21 ± 1.34 | 10.31 ± 1.24 |
| RvE2 | 333 | 159 | 10.48 ± 0.46 | 12.02 ± 0.72 |
| RvE3 | 333 | 201 | 15.49 ± 0.32 | 13.11 ± 1.59 |
|  |  |  |  |  |
| AA bioactive metabolome |  |  |  |  |
| LXA_4_ | 351 | 115 | 0.32 ± 0.10 | 0.30 ± 0.22 |
| LXB_4_ | 351 | 221 | 0.46 ± 0.56 | 0.98 ± 0.11 |
| 5S, 15S-diHETE | 335 | 235 | 86.44 ± 7.96 | 73.95 ± 1.59 |
| 15-epi- LXA_4_ | 351 | 115 | 5.25 ± 0.10 | 4.90 ± 0.57 |
| 15-epi- LXB_4_ | 351 | 221 | 4.59 ± 0.77 | 4.75 ± 1.28 |
| 13,14-dihydro-15-  oxo-LXA_4_ | 351 | 115 | - | - |
| 15-oxo-LXA_4_ | 349 | 115 | 0.06 ± 0.07 | - |
|  |  |  |  |  |
| LTB_4_ | 335 | 195 | 1.59 ± 0.25 | 1.50 ± 0.16 |
| 5S, 12S-fiHETE | 335 | 195 | 5.35 ± 0.22 | 4.65 ± 0.14 |
| ∆6-trans- LTB_4_ | 335 | 195 | 1.55 ± 0.08 | 1.50 ± 0.10 |
| 12-epi,  ∆6-trans- LTB_4_ | 335 | 195 | 1.54 ± 0.05 | 1.47 ± 0.11 |
| 20-OH-LTB_4_ | 351 | 195 | - | - |
|  |  |  |  |  |
| PGD_2_ | 351 | 189 | 17.10 ± 0.91 | 16.68 ± 2.53 |
| PGD_2_ | 351 | 189 | 6.68 ± 0.49 | 6.98 ± 0.12 |
| PGD_2_ | 351 | 193 | 13.21 ± 2.07 | 10.55 ± 0.54 |
| TXB_2_ | 369 | 169 | 51.90 ± 11.27 | 83.99 ± 11.29* |

**Table S1. Lipid mediator profiling of DFO-treated cDCs.**

BM-cDCs were incubated with DFO or vehicle control. After overnight culture, cells were quenched with ice-cold methanol and lipid mediators identified using LC/MS-MS based profiling. Results are expressed as pg/10^6^ and represent mean ± SEM. Below detection (0.1 pg) is identified as -. Q1, M-H (parent ion); and Q3, diagnostic ion in the MS-MS (daughter ion). * p< 0.05, ** p< 0.01 using Mann Whitney test.

**Figure S4. Unprocessed blots.** Uncropped blot for (a) Figure 1A, (b) Figure 3A and (c) Figure S1A.
